# The Management of Fevers in Germany

**Published:** 1878-08

**Authors:** W. S. Caldwell

**Affiliations:** Vienna, Austria


### Correspondence.

### THE MANAGEMENT OF FEVERS IN GERMANY.

By W. S. Caldwell, M. D., Vienna, Austria.

In the following article I may not be able to advance any new ideas upon the subject of the management of the febrile state. I propose, however, to discuss certain methods of procedure which are regarded by most practitioners of medicine as either too impracticable for adoption in private practice, or as resting upon evidence in point of utility which is not quite trustworthy.

So much, in fact, has been written, of late, on the cold water treatment of typhoid fever, that the subject would seem to be well nigh exhausted; and the only apology that I have to offer for this encroachment upon an already fully occupied field, is that I hope that some of the suggestions I shall make will be more easily carried out in private practice than those usually given by men who have at their command the abundant resources of a well directed hospital.

Much, however, as this subject has been discussed, and in spite of the wonderful statistics lately published by Liebermeister, showing the beneficial effects of the cold bath as an antipyretic agent, during my late visits to the English and French hospitals I found that typhoid fever was *generally* treated by the administration of the mineral acids, much after the plan that I was taught in the first lectures I attended a quarter of a century ago. In a course of lectures delivered by the professor of practical medicine in one of the New York colleges during the last winter on the same subject, he never even referred to the benefits to be derived from the use of the cold bath in the management of this disease.

A late work on the nature and management of fevers (Ueber Wesen und Behandlung des Fiebers), by Dr. Carl Emil Buss, of Bâle, is now attracting considerable attention in Germany.

The writer is a warm advocate of the fermentation theory as a cause of the febrile state, and he says that the irritant is a living organism, which, by its multiplication in the circulation, calls forth the lesion in each particular case.

"Others again claim that the presence of a living organism in the blood is not the *prime* cause of the complex symptoms, but that the products of decomposition which are produced by them, are more likely to act as an irritant, and thus cause the rise in temperature."

The injection of putrid matters into the circulation is found, he says, to produce septic fever in a direct ratio to the quantity of bacteria which such fluid contains. One of the most pernicious results that follow as a sequel of fevers, is a degeneration of the muscular substance of the heart, and a parenchymatous change in other organs essential to life. "Although hyperpyrexia may account in part for these results, it is probable that they are also largely produced by the presence of living animalaculæ, which as yet we are unable to discern, but which act essentially the same as the trichina spiralis upon the muscles of the body when it has gained access to the system."

Taking this view of the nature of fevers in general, which the author claims are, in their manifestations, essentially the same, our remedies should be such as have a tendency to destroy these low forms of animal life. "It is wonderful that those remedies which are the most powerful anti-pyretics, all possess in an eminent degree antiseptic properties also. In confirmation of the above theory, it is found that the remedies that act so happily in reducing animal heat when we have a high temperature, produce little or no effect when given to a healthy individual. It is very probable that the anti-pyretic properties of these drugs are effected by their combination within the circulation with the septic element that produced the fever, and in this way we have a favorable result produced."

While the author is a warm advocate of the use of cold baths to reduce the temperature in fevers, he thinks that their use should be supplemented by other treatment, for in such diseases as diphtheria, scarlatina, and the worst forms of abdominal typhus, our patient is stricken down at once, and succumbs rapidly to the overpowering effects of a prime cause, that our symptomatic treatment fails to reach.

The indications in the treatment of fevers are, first, to neutralize the "causal irritant;" and, second, to use such other means as we have at our command, to assist in the reduction of the temperature.

To fulfill the first indication, the best remedies are the salicylates and cresotinic acid. Buss says, "the antipyretic effects of the salicylate of soda cannot be equalled by any other drug. It is far preferable to the salts of cinchona, because it does not retard the elimination of carbonic acid in the system, and acts more energetically in reducing the animal heat. Within three or four hours after the drug was given, the fall of temperature was generally from two to three degrees, but when given largely, the reduction was as much as five degrees. This formula for the use of this remedy in typhoid fever is as follows:

| | |
|---|---|
| Salicylate of soda | 15 |
| Syrup of cinnamon | 30 |
| Water | 180 |

Mix.

Give one tablespoonful every three hours, excepting eight hours of the night, when the patient is allowed to sleep.

Should this dose offend the stomach, he advises to give one-third the quantity and repeat the dose every hour.

Cresotinic acid, given to the extent of 10 to 15 grams in the twenty-four hours, was found equally efficacious.

The effect of these remedies was, as the author asserts, to shorten the duration of typhoid fever. Their internal use was in most cases supplemented by the use of the cold bath, but as this part of the subject is not very different from that given by Liebermeister, I will not quote the latter in this connection.

The tabulated statistics that are given, showing the anti-pyretic effects of these remedies, I shall also omit.

PNEUMONIA IN CHILDREN.

Dr. Monti, a docent of the University of Vienna, a lecturer on diseases of children at the Poliklinik, and one of the very best teachers in this city, treats both catarrhal and croupous forms of pneumonia of children by giving to a child, one year old, teaspoonful doses of a one per cent. solution of the salicylate of soda every two hours. He envelops, at the same time, the chest and abdomen in large clothes wrung out of water of a temperature of 18.3° C., and changes these every one-half to two hours, according to the temperature. Expectorants of all kinds, he thinks are injurious, so long as the air vesicles are involved, and only uses them when the vesicles are no longer involved, and there is left simply bronchial irritation, with dryness of the mucous lining. In the

TYPHOID FEVER OF CHILDREN

he gives, to a child six years of age, a teaspoonful every three hours of a three per cent. solution of the salicylate of soda.

If the temperature does not run higher than 38.3° C., he uses no other treatment except nourishment in a fluid form, and red claret wine in doses according to the tendency that exists to a failure of the heart's action. Whenever the thermometer in the axilla registers a temperature above 38.3° C., he then resorts to the use of cold packing with water ranging from 13° C. to 18° C. If the case be a more severe one, and the temperature runs as high as 40° C. to 40.5° C., he resorts to cold baths, putting the patient into water at 30° C., and then adding cold water to reduce it from 18° to 24° C. These baths are only used as long as the fever is very high, the patient being kept in them from 10 to 30 minutes, according to the time necessary to reduce the temperature.

Speaking of the effects of quinia in fevers (upon which subject the doctor remarked that the average physician was a maniac), he only gives the remedy in their later stages, and then in small doses as a tonic.

To illustrate in what manner quinia acts as an anti-pyretic, he related the following case: "I was called a few days ago in consultation with another physician to see a child aged ten years, suffering from typhus abdominalis. As the attending physician was fond of giving quinia in this disease, I finally consented that the patient might have 6 decigrams of the drug, divided into three doses, and given at intervals of three hours, just before the exacerbation of the fever. The physician not understanding the metric system of weights (it having been introduced into Austria for only a few years), wrote his prescription for 6 *grams* instead of *decigrams*." Dr. Monti was called again soon after the child had taken the last dose, and found it as deaf as an adder, breathing heavily, and capable of being aroused only to semi-consciousness with great difficulty. The temperature was reduced to 35.5° C. in the axilla, though before the remedy was taken it was 40° C. Stimulants were given, cold was applied to the head, and warm applications to the feet, and after some hours the patient recovered from the effects of the drug. In this case, although there was such a marked reduction of temperature at the time, the following day the exacerbation of the fever was the same as before the remedy was given. The inference drawn by Dr. Monti from this case, as well as from an extensive trial of the drug in other cases of typhoid fever, is that quinia only acts as a *decided* anti-pyretic when given in doses that must be considered as *poisonous* in size, and that even when these doses are given daily, they exercise no influence in shortening the duration of the attack.

As bearing on this question of the anti-pyretic effects of quinia in this disease, I will give the following case from my own private note-book. During the fall of 1875, while we were having a severe epidemic of typhoid fever in Warren, Ills., I was called to attend the daughter, aged 13 years, of Mr. H——, a lawyer of that town.

The patient had enjoyed previous good health, and was large and well developed for her age.

The attack was severe from the outset, the temperature running to 40° C. after the first week, and rising to 40.5° C. after the middle of second, and was accompanied by marked cerebral disturbance. My plan has always been to combat vigorously a temperature that reaches or excels 40° C. For this purpose I usually wring cloths from water varying from 15° to 20° C., and envelop the body from under the axilla to the knees.

If the patient be an adult, the cloth used should be as large as a common sheet.

I direct that these cloths should be changed as often as every 15 to 30 minutes, until the temperature is brought down to a point that I consider compatible with the safety of the patient, say to 38° or 39° C.

To use this remedy requires considerable judgment on the part of the nurse, with whom I always leave a thermometer, so that my cold applications may be used intelligently.

As sometimes happens, the case above referred to was very sensitive to the application of the wet pack, and so after trying it in a rather unsatisfactory manner for a week, I determined to discontinue its use, and resort to quinia to fulfill the indications for which I had been using the cold water.

With this view I gave her 0.48 gram of the remedy at 4, 6 and 8 o'clock p. m., for three days in succession. The effect of the drug was to increase the nervous disturbance with my patient, without in the least lessening the temperature of the body, and finding her in every way worse at the expiration of this time, I fell back with renewed energy on my hydropathic treatment of the case.

From this time on I gave no medicine save a single dose of chloral-hydrate at night, kept the temperature down to 38.3° C., and my patient grew gradually better and made a good recovery, after a duration of the fever of eighteen days.

In Prof. Duchek's wards here in Vienna, to which, by the courtesy of his first assistant, Dr. Brenner, I have had free access at all times, the treatment of the milder cases of typhoid fever is entirely expectant.

The patient is given small doses of dilute sulphuric acid, but as this is their universally prescribed *placebo*, it must not be considered in the light of an attempt at medication.

One point in their dietetic rules here is worthy of note, and that is that no patient who has typhoid fever of even the mildest type, is allowed a single mouthful of solid food as long as he has any increase of temperature. Milk, soups and claret wine are used exclusively as nourishment.

When the case is more severe, and any treatment is considered necessary, either the salicylate of soda or quinia is given.

The former is preferred in the earlier stages of the disease, or where there exists any marked tendency to cerebral hyperæmia.

The manner of prescribing the remedy is as follows:

| | |
|---|---|
| Salicylate of soda | 5 |
| Syrup of raspberry | 20 |
| Water | 200 |

Mix.

Give one tablespoonful every three hours during the waking hours of the patient, or from 14 to 16 hours out of the twenty-four.

When quinia is used, 1 gram is given in the twenty-four hours, usually in three doses, at 2, 5 and 8 p. m.

Cathartics are not given, the bowels being moved every three or four days by injections.

But it is to the manner of using cold water in these wards that I wish to call the attention of the reader, as it is entirely practicable, and its value such as after considerable experience I have verified in my own practice.

First, *baths* are entirely ignored.

The arguments against them, are: first, that their use involves an amount of discomfort to the patient, that is not compensated for by any additional advantage that they offer over the wet sheet. Second, in using them it is necessary to subject the patient to an amount of physical exertion that is likely to act deleteriously on the future course of the disease. In other words, they deem it essential that the patient should be kept as absolutely quiet as possible during the whole course of the fever.

If he be feeble, in the later stages of the disease, he is not allowed to rise upright in bed, even to evacuate the bladder or bowels.

This perfect quiet is supposed to retard the fatty metamorphosis in the muscular tissue of the heart and other organs.

Fever patients here are usually dressed in a single garment, open in front, reaching to the feet, somewhat after the fashion of a lady's night-dress. A rubber sheet is put over the mattress to prevent its becoming damp. When the temperature rises to a point that is thought to be unsafe, they resort to the cold pack, and use it in the following manner. The patient, being dressed as above indicated, a sheet is wrung out of water of a temperature varying from 7.5° to 15.5° C., and in this his entire body is enveloped, and over this is thrown a dry covering. These sheets are changed every 10 to 20 minutes for from two to five times, a thermometer being kept constantly in the axilla to denote the fall in temperature that is required.

After the temperature has fallen, the patient's garment as well as his bedding is changed, for the sake of dryness.

By the assistance of two intelligent nurses, all this is accomplished with the least possible amount of exertion on the part of the patient.

If he be decidedly feeble, he is given either brandy alone, or milk punch before the wet sheets are used.

The advantage of the procedure above given is that it can be carried out easily in private practice, which is not the case with the cold baths as recommended by Liebermeister.

### Pneumonia.

To illustrate the treatment of this disease here in Vienna, I will give the clinical history of the following case, which I watched carefully every day during its progress:

Anna H., a domestic, aged 24 years, was admitted to the Allgemeinen Krankenhaus, May 13th, 1878. Three days before, the patient was taken suddenly ill, her first symptoms being a well marked chill, which was soon followed by a severe pain in the right side. On admission, the right lung was filled with an infiltration, she was raising rusty sputa, and had all the symptoms of a well-defined case of pleuro-pneumonia dextra.

Her respiration was 24, pulse 100, temperature 39.4° C.

Cloths wrung out of ice water were applied to the right side.

Internally, she was given one gram of the salicylate of soda every four hours, each dose being immediately followed by a large draught of cold water. She was also given an infusion of digitalis, made by adding 1 gram of the leaves of the herb, to 200 grams of boiling water, of which one tablespoonful was administered every four hours.

From the 13th to the 16th the case remained nearly stationary; the pleuritic pains in the right side were very severe, but the relief obtained by the application of the ice water was very marked indeed.

May 17th. Patient much worse.

Respirations, 45; pulse, 120; temperature, 40.5° C.

Physical examination showed the entire lower lobe of the left lung involved in a pneumonic process in the first stage, accompanied by a severe pleuritic pain in the same region.

May 18th. Patient still worse.

The lower lobe of the left lung was consolidated, and the upper lobe involved. Delirium, pulse 130, respiration 50, temperature 40.7° C. Digitalis and salicylate of soda were discontinued, and in their stead, quinia 30 Ctg. were given every six hours, combined with the free use of brandy. The entire chest, from under the axilla to below the ribs, was enveloped in wet cloths wrung from ice water which were changed every 20 to 40 minutes, depending upon the ease and tranquillity of the patient, as she called for them to be repeated as soon as they became warm.

May 19th. Respirations, 50; temperature, 40° C.; pulse, 125; patient still delirious. Prof. Duchek, who examined the case, and made it the subject of a clinical lecture, considered the prognosis as extremely grave, the only favorable symptom being a slight subcrepitant râle to be heard in the lower lobe of the right lung, showing a tendency to resolution. Patient was ordered increased dose of brandy, so that from 400 to 500 Gms. were to be given in the twenty four hours. The cold applications to the chest were vigorously continued.

May 20th. Patient better; temperature, 39° C.; pulse, 115; respiration, 25.

Stimulants lessened in quantity, cold to the chest hourly.

May 21st. Right lung nearly freed from exudation.

On account of the late involvement of the left lung, the patient had some fever until the 24th, from which date she rapidly convalesced.

I find here, both in fevers and in cardiac diseases, that digitalis is used only while there is a comparative vigorous circulation; in fact, to fulfill indications exactly opposite to those for which it is given by most physicians in the United States.

They use no counter-irritants to the chest in the hospital here, in cases of pleuro-pneumonia, but depend upon the cold cloths exclusively.

Expectorants are ignored, except to relieve a bronchial catarrh that may follow the lung complication as a sequel.

The favorite expectorant is a combination of sulphuric æther, syrup of liquorice and ipecac.

